# Lateral thinking in syndromic congenital cardiovascular disease

**DOI:** 10.1242/dmm.049735

**Published:** 2023-05-03

**Authors:** Agnese Kocere, Robert L. Lalonde, Christian Mosimann, Alexa Burger

**Affiliations:** ^1^ University of Colorado School of Medicine, Anschutz Medical Campus, Department of Pediatrics, Section of Developmental Biology, Aurora, CO 80045, USA; ^2^ Department of Molecular Life Science, University of Zurich, 8057 Zurich, Switzerland

**Keywords:** Congenital heart disease, Heart development, Mesoderm, Lateral plate mesoderm, Rare diseases, Embryo patterning, Animal models, Genotype-phenotype correlation, Cell fate, Cardiopharyngeal field, Hematopoiesis

## Abstract

Syndromic birth defects are rare diseases that can present with seemingly pleiotropic comorbidities. Prime examples are rare congenital heart and cardiovascular anomalies that can be accompanied by forelimb defects, kidney disorders and more. Whether such multi-organ defects share a developmental link remains a key question with relevance to the diagnosis, therapeutic intervention and long-term care of affected patients. The heart, endothelial and blood lineages develop together from the lateral plate mesoderm (LPM), which also harbors the progenitor cells for limb connective tissue, kidneys, mesothelia and smooth muscle. This developmental plasticity of the LPM, which founds on multi-lineage progenitor cells and shared transcription factor expression across different descendant lineages, has the potential to explain the seemingly disparate syndromic defects in rare congenital diseases. Combining patient genome-sequencing data with model organism studies has already provided a wealth of insights into complex LPM-associated birth defects, such as heart-hand syndromes. Here, we summarize developmental and known disease-causing mechanisms in early LPM patterning, address how defects in these processes drive multi-organ comorbidities, and outline how several cardiovascular and hematopoietic birth defects with complex comorbidities may be LPM-associated diseases. We also discuss strategies to integrate patient sequencing, data-aggregating resources and model organism studies to mechanistically decode congenital defects, including potentially LPM-associated orphan diseases. Eventually, linking complex congenital phenotypes to a common LPM origin provides a framework to discover developmental mechanisms and to anticipate comorbidities in congenital diseases affecting the cardiovascular system and beyond.

## Teaser

**Summary:** We conceptualize how phenotypes of syndromic congenital diseases affecting the cardiovascular system link to disrupted mechanisms in shared progenitor cells and developmental programs in the lateral plate mesoderm.

## Introduction

Comparative observations in different model organisms guide our understanding of how the human body develops, functions and manifests congenital anomalies. Numerous rare congenital diseases present with complex syndromic phenotypes that affect seemingly disconnected organs or cell types. Studying such disease phenotypes in model organisms can (1) reveal new, clinically meaningful insights about a potentially shared embryonic origin or molecular mechanism, and (2) inform about possible additional phenotypes in affected patients. However, decoding the underlying developmental and molecular causes of complex syndromic birth defects remains challenging when we lack a unifying concept to connect the observed phenotypes.

**Fig. 1.**
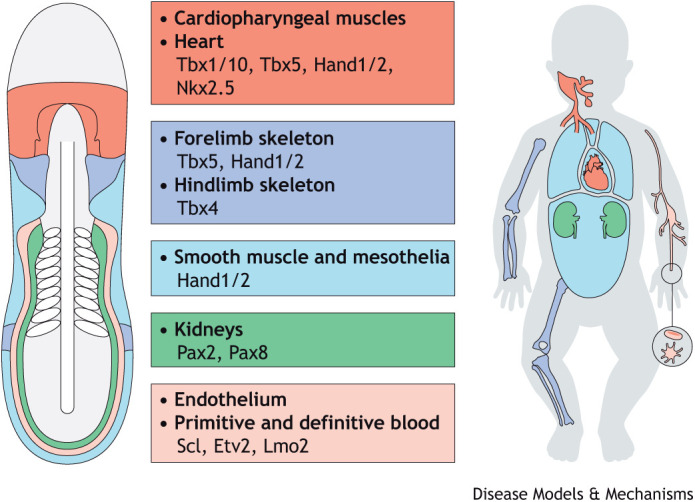
**The lateral plate mesoderm (LPM) develops into a dispersed set of organs that share a common embryonic origin.** Major LPM progenitor cell populations and their main transcription factors (middle), and their corresponding arrangement in a schematized vertebrate embryo during segmentation stages (left). The human infant (right) depicts the corresponding final organs and cell types with their distribution in the body.

**Fig. 2.**
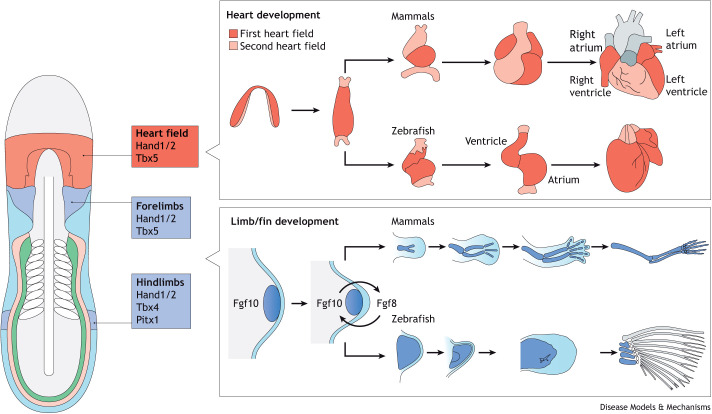
**Heart and limb develop from neighboring LPM populations with shared transcription factors.** The heart and the forelimb progenitors emerge within the anterior LPM in close proximity to each other, as depicted in a schematic representing the vertebrate embryo (left). On the right, we depict heart and limb/fin development in the mammalian and zebrafish embryo; the developmental process in zebrafish is similar to that in mammals. The heart forms by medial progenitor migration and leftward jog, resulting in the primitive heart tube that then undergoes complex remodeling to form a multi-chambered heart (top). The limb buds for both forelimbs and hindlimbs emerge from dedicated territories within the LPM via a conserved cascade of signaling events; interaction with the apical ectodermal ridge through Fgf ligand secretion supports limb bud outgrowth followed by LPM-based formation of connective tissue and skeletal structures (bottom).

Heart formation provides a powerful example for the close developmental and genetic relationship of embryonic lineages with seemingly distant locations in the final body plan (Bulatovic et al., 2016; Diogo et al., 2015; Meilhac and Buckingham, 2018; Stainier et al., 1993). The heart develops from the lateral plate mesoderm (LPM) that initially emerges at the edge of the forming vertebrate embryo (Meilhac and Buckingham, 2018; Prummel et al., 2020; Saint-Jeannet et al., 1992; Stainier et al., 1995) (Fig. 1). Curiously, the emerging heart field is part of a wider progenitor cell population called the cardiopharyngeal field (CPF) that includes progenitor cells forming various muscle groups of the neck and the head (Figs 1 and 2) (Chan et al., 2016; Diogo et al., 2015; Gopalakrishnan et al., 2015; Lescroart et al., 2015, 2021; Stolfi et al., 2010). In addition to the heart, the LPM also gives rise to several smooth muscle lineages, ventral dermis, mesothelia and connective tissue of the limbs, including long bones (Lane and Smith, 1999; Selleck and Stern, 1991; Warga and Nüsslein-Volhard, 1999) (Fig. 1). The kidneys, traditionally described as intermediate mesoderm in birds and mammals, share clonal and gene expression relationships with other LPM-derived progenitors across vertebrates (Fig. 1) (Davidson et al., 2019; Mansour et al., 2022; Mattonet et al., 2022; Takasato and Little, 2015; Takasato et al., 2014). This complex developmental and genetic architecture renders the LPM sensitive to defects in its patterning and cell fate specification, with broad consequences for several of its descendant organs and tissues. Case in point, heart-hand syndromes manifest as cardiovascular and forelimb anomalies (Camarata et al., 2010; Yin et al., 2018, 2020). Although these phenotypes present in seemingly distant organs, the progenitors for both the heart and forelimb skeleton share a close developmental connection as they emerge from adjacent cell territories within the LPM, which also have a considerable number of co-expressed genes (Figs 1 and 2) (Mori and Bruneau, 2004; Silverman and Hurst, 1968; Tamari and Goodman, 1974; Yin et al., 2020).

Overlaying complex syndromic disease phenotypes with developmental information provides a conceptual framework to discover underlying mechanisms and possible comorbidities relevant to long-term patient health. Here, we discuss the LPM as a developmental concept to connect seemingly disparate phenotypes in human birth defects affecting the heart and beyond with known and unknown causative genes. We further present as-of-yet orphan syndromes missing causative gene associations that manifest as potential LPM diseases due to their affecting two or more LPM-derived tissues. We lastly outline how clinical, computational and developmental biology laboratories can productively collaborate towards revealing the causes of complex congenital syndromes affecting the heart and comorbidities.

## The LPM as context for cardiovascular system development

LPM formation and patterning is regulated by deeply conserved principles across chordates (Box 1) (Prummel et al., 2020). Its emergence is closely tied to dorsal-ventral patterning of the vertebrate embryo, which is driven by secreted Bmp and Nodal signaling molecules that control downstream Smad transcription factors (Graff et al., 1996; Liu et al., 1996; Müller et al., 1999; Savage et al., 1996; Schier and Talbot, 2005; Sekelsky et al., 1995; Shi et al., 1997; Zhang et al., 1996). While Bmp regulates the dorsal-ventral arrangement of the body axis, Nodal activity additionally supports ectoderm versus mesendoderm patterning (Conlon et al., 1991; Davidson et al., 2015; Gritsman et al., 1999; Hayes et al., 2021; Kattman et al., 2011; Martyn et al., 2019; Schier and Talbot, 2005; Zhou et al., 1993). During gastrulation, the LPM emerges as ventral and lateral mesoderm territory (Garcia-Martinez and Schoenwolf, 1993; Gurdon, 1995; Lane and Smith, 1999; Mead et al., 1996; Selleck and Stern, 1991). In zebrafish, Smad proteins cooperate with Eomesa, Foxh1 and Mixl1 to induce the LPM, yet whether this combination is necessary and sufficient across vertebrates remains to be determined (Bjornson et al., 2005; Prummel et al., 2019; Slagle et al., 2011). Nonetheless, the basic concept of ventral LPM-inducing activity seems to be conserved across chordates and sets up the emerging LPM as a dedicated progenitor field for its various downstream cell fates (Figs 1 and 2, Box 1) (Prummel et al., 2019, 2020).

**Box 1. Evolutionary context of lateral plate mesoderm (LPM) and cardiovascular fate formation**

Despite a wealth of data on downstream fates, little is known about how the LPM partitions into its individual territories and how these relate to each other. Our current insights suggest that LPM-derived organs such as the heart evolved by integrating LPM-focused regulatory programs and cell fate mechanisms that became refined from pre-existing larger cell fields with as-of-yet unknown purpose (Kaplan et al., 2015; Monahan-Earley et al., 2013; Onimaru et al., 2011; Prummel et al., 2020; Tanaka, 2016) (Fig. 1). The seemingly disconnected functions and distributions of downstream fates emerging from the LPM have rendered the study of their joint origins challenging. Comparative studies involving distantly related chordate species now highlight the LPM as a fertile ground for evolutionary innovations that have contributed to critical aspects of vertebrates, including, but not limited to, head and neck muscles for feeding, appendages for locomotion and chambered hearts for increased metabolic needs (Ericsson et al., 2013; Onimaru et al., 2011; Prummel et al., 2019; Stephenson et al., 2017; Yong et al., 2021). In particular, the evolutionary context of heart development has advanced our understanding of cardiovascular development and connections to disease.

The forelimb and hindlimb skeleton are the most diverged, non-obvious LPM derivatives. Decades of phenotypic, genetic and molecular work across model systems and in human congenital disease have uncovered that both heart and forelimb progenitors express Tbx5, Hand2 and other transcription factors (Rallis et al., 2003; Steimle and Moskowitz, 2017). Further, both heart and limb progenitor fields form adjacent to each other in the LPM (Fig. 1 and 2). Zebrafish and mouse data propose that restriction of the cardiac mesoderm by retinoic acid is required for the initiation of the limb field (Tanaka, 2016; Waxman et al., 2008; Zhao et al., 2009). Zebrafish and mouse *raldh2* (*aldh1a2*)*/Raldh2* (*Aldh1a2*) mutants lack anterior paired appendages, and retinoic acid inhibition in zebrafish induces posterior expansion of the cardiac field correlating with failed fin bud initiation (Gibert et al., 2006; Grandel et al., 2002; Waxman et al., 2008). These phenotypes suggest that the regionalization of the LPM into cardiac mesoderm and more posterior derivatives contributed to forelimb field evolution. Analysis of *Nkx2.5* expression dynamics documented that this regionalization occurs during lamprey development, but not in amphioxus, suggesting that this process evolved in the vertebrate family branch (Onimaru et al., 2011). The *Tbx5* expression domain is defined by nested Hox expression, specifically at the anterior borders of the *Hox5*- and *Hox6*-expressing LPM (Minguillon et al., 2012). Although many questions regarding the evolutionary and developmental relationship of heart and forelimb mesoderm remain unresolved, experimental evidence suggests that the evolution of the forelimb field followed an anterior restriction of the cardiac mesoderm. However, the initial purpose of this restriction remains debated; forelimb origins have been suggested to have started as the seventh pharyngeal arch or as part of broader models of fin formation along the body axis, yet both possibilities await further data (Diogo, 2020; Sleight and Gillis, 2020; Tulenko et al., 2013).

The finding that second heart field progenitors share a clonal origin with craniofacial muscles has revealed another unanticipated developmental and evolutionary connection – the cardiopharyngeal field (CPF). The combined CPF is defined by expression of *Tbx1*, *Nkx2.5* and others. Subsequent work across models, including the chordate *Ciona*, has revealed deeply conserved gene regulatory mechanisms within this intertwined progenitor field (Diogo et al., 2015; Stolfi et al., 2010; Wang et al., 2013) (Fig. 2). However, as with the heart and limb connection, the original purpose of an ancestral CPF remains a mystery. The cardiopharyngeal progenitors forming heart and head muscles are hypothesized to employ separately evolved, but functionally convergent, striated muscle programs (Diogo et al., 2015; Steinmetz et al., 2017). Over the course of chordate head evolution, cardiopharyngeal progenitor cells are thought to have acquired the ability to self-renew prior to differentiating into either heart or branchiomeric (head/neck) muscle groups and to respond to appropriate signals to specialize in different locations (Diogo et al., 2015; Kaplan et al., 2015; Tolkin and Christiaen, 2012). Evolutionary and comparative studies pointed out that the emergence of heterogeneous branchiomeric muscle groups coincides with the appearance of chambered hearts in stem vertebrates, suggesting a shared developmental and evolutionary history (Diogo et al., 2015; Moorman and Christoffels, 2003; Simões-Costa et al., 2005). In tunicates, trunk ventral cells are multipotent cardiopharyngeal progenitors that contribute to the heart and pharyngeal muscle groups (Kaplan et al., 2015; Stolfi et al., 2010; Wang et al., 2017b).

These LPM-associated evolutionary puzzles highlight the power of comparative, cross-species studies to start defining the cellular and molecular framework that govern the context of heart development. Combining developmental and evolutionary developmental biology (evo-devo) as comparative approaches with genetic insights from human disease has strong potential to advance our knowledge in all these disciplines.

A hallmark of LPM architecture is the shared expression of transcription factor genes across seemingly disparate lineage progenitors; whether this shared expression reflects a clonal origin of co-expressing progenitors or co-opted gene regulation for similar regulatory purposes remains a major open question. In the emerging heart field, Tbx5 synergizes with Nkx2.5 (Nkx2-5), Gata4, Hand1/2 and other cardiac transcription factors to regulate heart tube precursors that develop from the LPM-embedded first and second heart fields (Greulich et al., 2011; Hiroi et al., 2001; Pi-Roig et al., 2014; Steimle and Moskowitz, 2017). The first heart field contains early-differentiating progenitors that form the primitive heart tube; descendant cells of the second heart field differentiate subsequently at both poles of the emerging heart tube (Dyer and Kirby, 2009; Laura et al., 2020; Rochais et al., 2009) (Figs 1 and 2). In the LPM immediately posterior to the emerging heart field, forelimb/pectoral fin bud initiation requires Tbx5 expression in the fin/limb-specifying field to induce Fgf10, which then activates Fgf8 in the overlying apical ectodermal ridge (Cunningham and Duester, 2015; Garrity et al., 2002; Minguillon et al., 2009, 2012; Moreau et al., 2019; Parrie et al., 2013; Rodriguez-Esteban et al., 1999; Takeuchi et al., 1999) (Fig. 2). Hand2 establishes the anterior-posterior forelimb axis through activating *Shh* and antagonizing *Gli3* (Charité et al., 2000; Galli et al., 2010; Osterwalder et al., 2014)*.* In the hindlimb-specifying field, Pitx1 activates Tbx4, which in turn induces Fgf10, achieving an analogous feedback loop with Fgf8 in the apical ectoderm ridge. Although forelimbs and hindlimbs display unique morphologies, Tbx4 can function in place of Tbx5 when expressed in the forelimb-specifying region (Don et al., 2016; Minguillon et al., 2005, 2012; Rodriguez-Esteban et al., 1999; Takeuchi et al., 1999) (Fig. 2). Despite their physical proximity and the fact that both the heart and the forelimb fields express Tbx5 and Hand2, it remains unclear whether these two lineages share a common progenitor or whether they arise from disparate progenitor fields (Ahn et al., 2002; Charité et al., 2000; Fernandez-Teran et al., 2000; Garrity et al., 2002; Rodriguez-Esteban et al., 1999; Steimle and Moskowitz, 2017; Yelon et al., 2000).

Expanding the developmental context of heart formation within the LPM, emerging heart progenitors are part of the CPF that expresses Tbx1 and Nkx2.5/7 and contributes to craniofacial muscles beyond the heart (Comai et al., 2019; Diogo et al., 2015; Lescroart et al., 2010; Meilhac et al., 2004; Nomaru et al., 2021; Xu et al., 2004; Zhou et al., 2008). Whether the entire CPF is one lineage field or a composite of the LPM and other anterior mesodermal lineages remains to be clarified (Figs 1 and 2). Nonetheless, the activities of Mesp1, Nkx2.5 and Tbx1/10, as well as their related gene-regulatory networks, indicate a homology between tunicate trunk ventral cells and the vertebrate CPF (Stolfi et al., 2010; Tolkin and Christiaen, 2012; Wang et al., 2013) (see also Box 1).

**Fig. 3.**
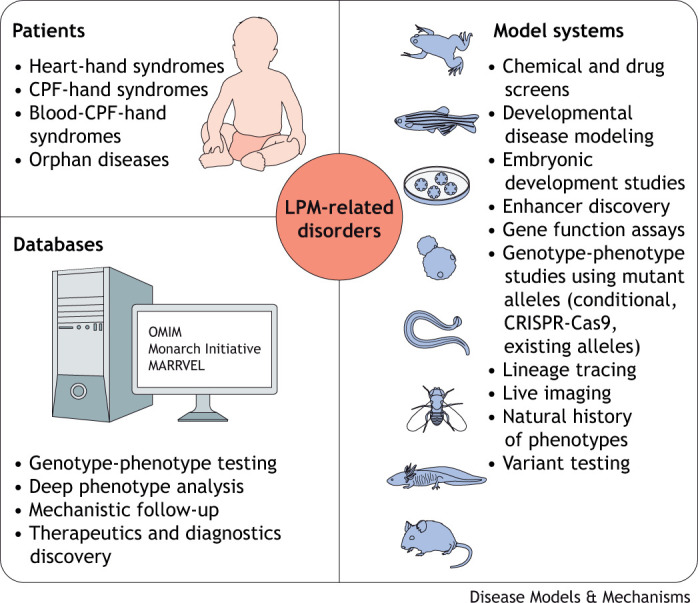
**Discovery of disease-causing genes and mechanisms.** Curated public databases assemble a wealth of patient-based sequencing data, genetic and phenotype information, and more. These data enable analyses of the intersection of diseases with related phenotypes and developmental similarities, such as LPM-centered anomalies. The developmental function of gene loss or gain of function, polymorphisms and other genetic changes are then testable in model systems, such as animal models that carry orthologs of human disease-associated human genes or patient-derived *in vitro* models. CPF, cardiopharyngeal field; LPM, lateral plate mesoderm; MARRVEL, Model organism Aggregated Resources for Rare Variant ExpLoration; OMIM, Online Mendelian Inheritance in Man.

During development, other LPM lineages intersect with cardiac precursors in origin, gene expression or both. The endocardium emerges from anterior *Scl/Tal1*- and *Etv2*-expressing progenitors that have a distinct morphology and migration path compared to that of other cranial endothelial cells (Bussmann et al., 2007; DeRuiter et al., 1992; Harris and Black, 2010; Ivanovitch et al., 2017; Misfeldt et al., 2009; Proulx et al., 2010) (Fig. 1). In the trunk, *Scl/Tal1*-expressing progenitors develop into trunk endothelium and first erythrocyte progenitors, which form the initial circulatory system components (De La Garza et al., 2019; Garcia-Martinez and Schoenwolf, 1992; Mead et al., 1996; Pardanaud et al., 1996; Stainier et al., 1996) (Figs 1 and 3). In parallel, *Pax2*- and *Pax8*-positive LPM progenitors form the kidney, as initially discovered by the contribution of Pax2/8 perturbation to kidney disease (Bouchard et al., 2002; Eccles et al., 2004; Keller et al., 1994; McMahon, 2016; Poleev et al., 1992; Torres et al., 1995) (Fig. 1). Recent work in zebrafish has documented that *pax2a*-expressing LPM progenitors contribute to endothelial and erythroid fates besides forming kidney lineages (Mattonet et al., 2022). The kidney precursors initially reside laterally to endothelial progenitors at least in the teleost LPM, contrary to in mammals, in which they reside between somites and endothelial precursors as ‘intermediate mesoderm’ (McMahon, 2016; Mullins et al., 1996; Perens et al., 2016; Takasato and Little, 2015; Terashima et al., 2014). These data suggest that kidney progenitors can be regarded as an LPM lineage. Lastly, the lateralmost LPM forms the *Hand2*-expressing mesothelium progenitors that surround internal organs and the body cavities, as well as different smooth muscle types (Ariza et al., 2016; Gays et al., 2017; Prummel et al., 2022; Shin et al., 2009). Work in chicken has documented interactions and stepwise separation between smooth muscles, blood and endothelial progenitors as driven by Hand2, Wnt, Bmp and Notch (Shin et al., 2009). Further underlining non-autonomous cross-talk between the stripes, loss of *hand2* in zebrafish perturbs the balance between endothelial progenitors and kidney formation (Perens et al., 2016, 2021). Subsequent blood flow has a major impact on tissue remodeling and even the emergence of key cell fates, as, after the onset of circulation, mechanical forces influence the budding of Runx1-expressing definitive hematopoietic stem cell progenitors from the ventral wall of the dorsal aorta (Boisset et al., 2010; Downing et al., 1993; Kissa and Herbomel, 2010; Lancino et al., 2018; Müller et al., 1994; North et al., 2009; Traver et al., 2003).

Taken together, the LPM harbors progenitor populations for multiple organs, providing a model to link perturbations in shared precursors to causation of congenital defects affecting two or more LPM-derived organs (Gays et al., 2017; Nishimoto and Logan, 2016; Prummel et al., 2019, 2020, 2022; Tanaka, 2016). Yet, the LPM is not of single clonal origin, and major organ precursors segregate as early as during gastrulation. Consequently, alternative mechanisms contributing to comorbidities could involve perturbed organ progenitor positioning, signaling interactions and shared gene expression across LPM domains. For this Review, we consider LPM-affecting diseases as those that affect two or more organs or cell types of LPM origin and/or that share LPM-centered gene expression profiles. The developmental and evolutionary peculiarities of the LPM underline how mutations in genes affecting common progenitors or shared regulatory mechanisms could trigger syndromic defects with complex comorbidities in seemingly unrelated organ systems.

## Syndromes with joint heart and limb anomalies

Joint heart and upper limb anomalies occur in several human congenital syndromes (Fig. 2). Affected patients share common clinical presentations that vary in their phenotypic expressivity, described under the umbrella term heart-hand or upper limb-cardiovascular syndromes. Although the genetic bases for several of these diseases have been uncovered (Table 1**,** Box 2), many remain without an assigned genetic cause (Table 2). These syndromes often present with additional respiratory, endocrine, metabolic, hematopoietic or neurologic symptoms for which clinical urgency can dominate over the heart and hand phenotypes, including symptoms that arise as complications from the primary cardiac defects (Metra et al., 2011; Tamari and Goodman, 1974) (Tables 1 and 2).

**Box 2. OMIM for diseases mentioned throughout the text resulting in a comparison table**

The table within the OMIM portal summarizes the causative genes and molecular bases of the syndrome as well as the inheritance pattern, if known, and several classes of phenotypes. This table was generated with the search query detailed in the supplementary information.

**Table 1.**
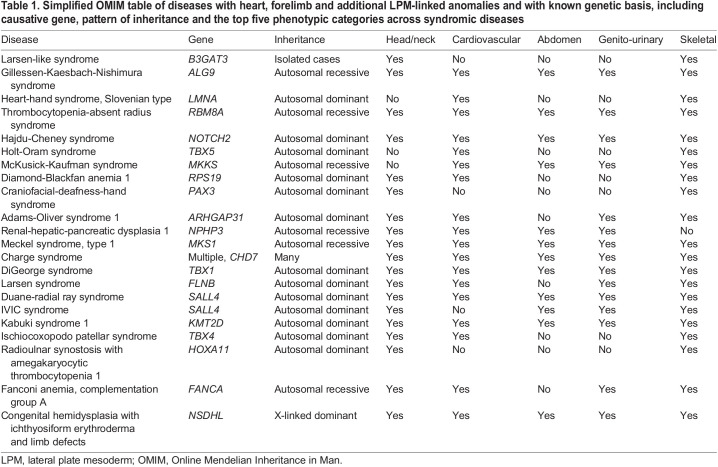
Simplified OMIM table of diseases with heart, forelimb and additional LPM-linked anomalies and with known genetic basis, including causative gene, pattern of inheritance and the top five phenotypic categories across syndromic diseases

**Table 2.**
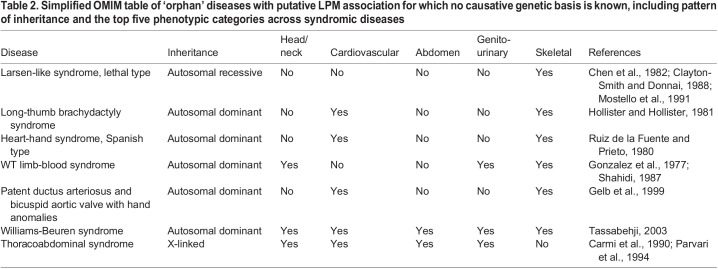
Simplified OMIM table of ‘orphan’ diseases with putative LPM association for which no causative genetic basis is known, including pattern of inheritance and the top five phenotypic categories across syndromic diseases

Holt-Oram syndrome is the prototypic heart-hand syndrome. It manifests as autosomal-dominant upper (anterior) limb and shoulder girdle abnormalities with a spectrum of congenital heart defects [Online Mendelian Inheritance in Man (OMIM) #142900] (Hurst et al., 1991; Temtamy, 1985). The most common presentation of Holt-Oram syndrome is triphalangeal thumbs and cardiac atrial septal defects, although the severities of limb and heart defects vary widely. In 1997, two separate groups confirmed that mutations in *TBX5* were causative for Holt-Oram syndrome, resulting in predicted instances of haploinsufficiency (Basson et al., 1997; Yi et al., 1997). Ever since linking *TBX5* mutations to the syndrome, a growing number of variant *TBX5* alleles have been uncovered in affected patients. Loss-of-function mutations or *TBX5*-spanning large deletions cause the most severe phenotypes (Basson et al., 1997; Gruenauer-Kloevekorn and Froster, 2003) (Borozdin et al., 2006). Missense variants may cause distinct phenotypes depending on the position of the mutation (Yang et al., 2000). The human syndromic phenotypes are recapitulated in recessive *Tbx5* loss-of-function mouse models (Bruneau et al., 2001; Rallis et al., 2003), and zebrafish *tbx5a* mutants and morphants have co-occurring heart and pectoral fin anomalies (Fig. 2, Table 3) (Ahn et al., 2002; Garrity et al., 2002). Select putative gain-of-function *TBX5* alleles cause atypical Holt-Oram syndrome with mild skeletal defects and paroxysmal atrial fibrillation (Postma et al., 2008). A 48 kb duplication encompassing *TBX5* exons 2 through 9 is associated with mild limb defects and non-septal cardiac defects (Patel et al., 2012).

**Table 3.**
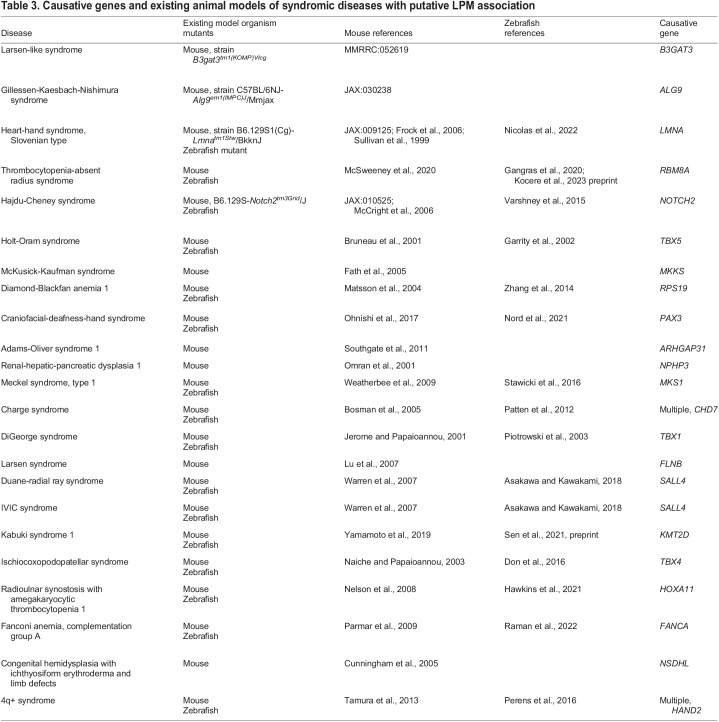
Causative genes and existing animal models of syndromic diseases with putative LPM association

Loss of the *Tbx5* paralog *Tbx4* in mice (Naiche and Papaioannou, 2003; Rodriguez-Esteban et al., 1999), and *tbx4* in zebrafish (Don et al., 2016), results in hindlimb or pelvic fin absence (Fig. 2, Table 3), although mutant mice do not survive embryonic stages due to fatal lung failure (Arora et al., 2012) (Table 3). This combination of developmental phenotypes links to *Tbx4* expression in the hindlimb bud, as well as in the future lung mesothelium that is likely to be of LPM origin. Curiously, dominant *TBX4* perturbations in humans largely cause cardiopulmonary defects, including pulmonary arterial hypertension and patent ductus arteriosus, in which the pulmonary circuit fails to remodel into a functional unit before birth (D'Alto and Mahadevan, 2012; Haarman et al., 2019; Haarman et al., 2020; Karolak et al., 2023; Welch and Chung, 2020; Yarboro et al., 2022). Patients with heterozygous *TBX4* variants have mild limb anomalies affecting the patella or joints (Haarman et al., 2019, 2020; Karolak et al., 2023). Notably, the human phenotypes affecting hindlimbs are distinct from those arising in mouse loss-of-function models, which seem to stem from possible species-specific partial compensation of Tbx4 by Pitx1 and from Isl1/Ldb function in murine hindlimb development (Duboc et al., 2021; Itou et al., 2012; Narkis et al., 2012) (Fig. 2).

Mutations in *HAND1* and *HAND2* have been associated with cardiac anomalies such as tetralogy of Fallot, hypoplastic left ventricle and others (Lu et al., 2016; Reamon-Buettner et al., 2008, 2009). These basic helix-loop-helix (bHLH) transcription factors have widespread expression in the early LPM and in the subsequent cardiac, pharyngeal arch, limb and mesothelial progenitors (Charité et al., 2000; Gibert et al., 2006; Prummel et al., 2022). Hand1 and Hand2 have unique as well as redundant functions in cardiac and limb patterning in mice, controlling epithelium formation, extracellular matrix composition, cell migration and the expression of downstream transcription factor genes (Barnes and Firulli, 2009; Barnes et al., 2010; Conway et al., 2010; Firulli et al., 1998, 2010). Curiously, zebrafish only retained *hand2* in their genome, and *hand2* mutants develop cardiac and branchial arch defects, hypoplastic pectoral fins and reduced mesothelium that results in herniation (Garavito-Aguilar et al., 2010; Hu et al., 2011; Perens et al., 2016; Prummel et al., 2022; Yelon and Stainier, 2005; Yelon et al., 2000). Compared to genes encoding other cardiac transcription factors, *HAND1/2* seem to be less frequently mutated in congenital disease, possibly due to their partial redundancy and potential to compensate for one another upon perturbation (Barnes et al., 2011; Charité et al., 2000; McFadden et al., 2005). Notably, partial trisomy of distal chromosome 4q (4q+; no OMIM reference), which contains the *HAND2* locus, is associated with cardiac, digit, kidney and craniofacial defects, indicating broader LPM perturbation during development (Battaglia et al., 2005; Lundin et al., 2002; Lurie, 2005). Elegant studies have linked mouse *Hand2* dosage to developmental phenotypes that recapitulate major aspects of human 4q+ syndrome (Table 3) (Tamura et al., 2013). Together, these shared heart and limb transcription factors provide clear examples for LPM-based comorbidities.

## Congenital craniofacial anomalies with cardiac comorbidities

Heart-hand syndromes feature obvious LPM-linked comorbidities, yet additional heart-affecting syndromes with complex comorbidities seem also to be influenced by shared LPM progenitors and/or shared genetic programs (Figs 1 and 2, Tables 1 and 2, Box 2). DiGeorge syndrome (OMIM #188400) is caused by autosomal-dominant 22q11.2 deletions (Wilson et al., 1993). Varying widely between patients, the most consistent symptoms of DiGeorge syndrome are hypocalcemia from parathyroid hypoplasia, thymus hypoplasia and T-cell deficiencies, submucosal cleft palate and cardiac outflow tract defects. Patients with 22q11.2 deletion can also present with variable craniofacial defects, encompassing conditions known as Shprintzen (velocardiofacial) syndrome or Takao syndrome, associated with dysphagia and a broad range of heart defects (Karpinski et al., 2022; Motahari et al., 2019; Shprintzen, 1994; Yagi et al., 2003; Yamagishi et al., 2016). Nonetheless, 22q11.2 harbors several genes and as-of-yet incompletely charted regulatory elements (Fernández et al., 2005; Saitta et al., 2004), and no single gene has been linked to the full phenotype spectrum. Consequently, DiGeorge syndrome could comprise multiple syndromes caused by individual genes in the 22q11.2 locus (Burn, 1999; Wilson et al., 1993).

The major phenotypes of DiGeorge syndrome can potentially be explained by *TBX1* haploinsufficiency, with other symptoms attributed to the cumulative effects from loss of other 22q11.2 genes (Jerome and Papaioannou, 2001; Lindsay et al., 2001; Paylor et al., 2006; Yagi et al., 2003). Notably, patients with point mutations in *TBX1* display characteristic phenotypes of 22q11.2 deletion patients (Yagi et al., 2003), although they do not develop the full spectrum of DiGeorge syndrome. Mice hemizygous for the highly conserved 22q11.2 region display mild cardiac outflow tract defects, as phenocopied in heterozygous *Tbx1* mutant mice (Lindsay et al., 1999, 2001). Homozygous *Tbx1* mutant mice (Jerome and Papaioannou, 2001; Merscher et al., 2001) and *tbx1* mutant zebrafish (*van gogh*, *vgo*) (Piotrowski et al., 2003) recapitulate the major structural defects observed in patients with severe DiGeorge syndrome, including heart, thymus and craniofacial defects (Table 3), but do not recapitulate all aspects of human DiGeorge syndrome. Notably, the expression pattern of Tbx1 is not single lineage or LPM specific, but rather a mix of different anterior/rostral cell types, including the LPM, neural crest and additional head mesoderm, that awaits concise definition. Consequently, untangling individual contributions of *Tbx1* function in individual cell types, as well as in cell-cell crosstalk, in head and heart structures remains an ongoing challenge in the field.

In addition, patients with atypical 22q11.2 microdeletions that leave *TBX1* unaffected still show craniofacial and cardiac defects, as well as velopharyngeal insufficiency (Shi and Wang, 2018; Verhagen et al., 2012). One possibility is that these patients lack *cis*-acting regulatory elements required for modulating *TBX1* expression; alternatively, deletion of *CRKL*, another 22q11.2 gene that has also been linked to craniofacial and heart anomalies in mouse models (Guris et al., 2001, 2006; Motahari et al., 2019; Verhagen et al., 2012), triggers the craniofacial and cardiac defects of DiGeorge syndrome. Further, modifying alleles outside the 22q11.2 microdeletion may synergize with *TBX1* to create unique, atypical phenotypic manifestations (Aggarwal and Morrow, 2008). Although DiGeorge syndrome is complex and incompletely understood, it provides a powerful example for the complex interplay of cell lineages associated with heart development across the LPM and beyond.

## Hematological disorders with complex comorbidities

Several predominantly hematopoietic disorders feature seemingly unrelated structural comorbidities (Tables 1 and 2, Box 2). A classic example is thrombocytopenia-absent radius (TAR) syndrome (OMIM #274000), a rare congenital disease affecting less than 1:100,000 live births. In its most selective manifestation, TAR syndrome patients feature a severely reduced number of platelet-forming megakaryocytes (<30×10^9^ platelets/l of blood), which correlates with hypo-megakaryocytic thrombocytopenia, and absent radius bones (Bonsi et al., 2009; Greenhalgh, 2002; Hall et al., 1969; Klopocki et al., 2007; Petit and Boussion, 2022; Thompson et al., 2001). The blood coagulation defect, which may be transient, is seen in all cases, and, in over 90% of patients, manifests as symptomatic within the first 4 months of life (Hall et al., 1969). Curiously, the coagulation defect resolves with increasing age, but it needs to be determined whether this is due to the migration of hematopoietic progenitors from the fetal liver to the fetal bone marrow. The non-hematopoietic syndromic phenotypes have variable expressivity, ranging from mild joint issues to absent radii, while several TAR syndrome patients also present with variable heart and kidney anomalies, intolerance to cow's milk presenting as persistent diarrhoea and failure to thrive, and can experience exacerbation of thrombocytopenia (Whitfield and Barr, 1976) later in life. More rarely, patients present with mild craniofacial anomalies. Genetically, TAR syndrome associates with a heterozygous 1q21.1 microdeletion removing at least ten genes, including the gene encoding the exon junction complex factor RBM8A/Y14 that ubiquitously contributes to the regulation of mRNA splicing, nonsense-mediated decay and mRNA localization (Gehring et al., 2005; Klopocki et al., 2007; Palacios et al., 2004). Hypomorphic perturbation of *RBM8A* is assumed to cause TAR syndrome, as the 1q21.1 microdeletion, combined with putative hypomorphic *RBM8A*/*Y14* alleles, has been found in most sequenced patients (Albers et al., 2012, 2013). However, the mechanistic link between hypomorphic *RBM8A* and the peculiar phenotype combination of TAR syndrome remains to be elucidated. Notably, TAR syndrome affects predominantly LPM-derived cell lineages in the blood, forelimb skeleton, heart and kidneys. Considering TAR syndrome an LPM-associated pathology might provide a framework to investigate its mechanistic underpinnings (Kocere et al., 2023 preprint) (Figs 1 and 3).

Another ultra-rare syndrome, autosomal-dominant radioulnar synostosis with amegakaryocytic thrombocytopenia (RUSAT1; OMIM #605432), features severe coagulation defects with restricted pronation-supination of the forearm (Thompson and Nguyen, 2000; Thompson et al., 2001). Two RUSAT1-affected families have been reported to carry mutations in *HOXA11*, a Hox gene involved in regional bone, muscle and tendon patterning (Hawkins et al., 2021; Swinehart et al., 2013); how *HOXA11* perturbation causes the hematopoietic defects remains unknown. By contrast, other RUSAT1 cases have been associated with mutations in the *MECOM* locus that spans the *MDS1* and *EVI1* genes (Niihori et al., 2015); the EVI1 transcription factor broadly controls cell cycle genes and is dynamically expressed in various tissues, including the forming limbs (Bard-Chapeau et al., 2012; Celá et al., 2013; Li et al., 2022; Nucifora, 1997). Notably, Evi1 is an upstream regulator of hematopoietic transcription factors, including the megakaryocyte-erythrocyte regulator Gfi1 (Ayoub et al., 2018; Chiba et al., 2020; Konantz et al., 2016).

In addition to TAR and RUSAT1, other rare hematopoietic disorders with co-occurring limb anomalies have been reported. Individuals with Fanconi anemia (OMIM #609054) can present with digit anomalies, such as unilateral radial anomalies that include duplicated thumbs (Bhandari et al., 2022; Tischkowitz and Hodgson, 2003). Other examples include WT limb-blood syndrome (OMIM #194350) (Gonzalez et al., 1977), Diamond-Blackfan anemia 3 (OMIM #610629) (Gazda et al., 2006; Landowski et al., 2013) and congenital hemidysplasia with ichthyosiform erythroderma and limb defects (OMIM #308050) (Happle et al., 1996). Notably, TAR, Fanconi anemia and Diamond-Blackfan anemia are seemingly caused by mutations in ubiquitous regulatory factors, suggesting an early developmental susceptibility to their activity levels that remains to be elucidated. Nonetheless, whether the root of these syndromic phenotypes is an earlier developmental LPM phenotype remains unclear, yet provides a working hypothesis to study their embryonic origins.

## Orphan diseases with potential LPM connections

In the examples above, we outlined the rationale for considering a shared LPM origin for syndromic congenital heart and cardiovascular diseases as part of complex syndromes with comorbidities affecting LPM-associated structures. A conceptual framework to connect syndromic phenotypes to a joint developmental basis extends to linking structural birth defects with fetal exposure to environmental factors such as teratogenic compounds (Box 3). Ultimately, combining syndromes with similar phenotypes via shared molecular pathways or developmental processes may shed light on orphan diseases with as-of-yet unknown causes and, at times, no clear all-encompassing diagnoses. Orphan diseases (Table 2, Box 2) remain understudied, as they are exceedingly rare, at times affecting single families or, alternatively, may have complex polygenic origins (Chen and Altman, 2017).

**Box 3. Environmental impact on heart and forelimb development**

Beyond syndromes with a trackable genetic basis, insights into developmental and genetic programs help explain the consequences of exposure to teratogenic agents. The most infamous example is thalidomide syndrome or embryopathy linked to the use of thalidomide (also known as α-phthalimidoglutarimide) to treat morning sickness in pregnancy (Donovan et al., 2018; Kim and Scialli, 2011; Rehman et al., 2011; Somers, 1960; Vargesson, 2015), which affected thousands of pregnancies in the late 1950s. Infants were born with severe limb defects (phocomelia), eye and ear deformities, and congenital heart defects, depending on the gestational age at exposure and duration of treatment (Donovan et al., 2018; Vargesson, 2015). We now know that thalidomide syndrome is caused by compound-promoted degradation of several zinc finger transcription factors, including SALL4 (Donovan et al., 2018). Consistent with this discovery, thalidomide syndrome phenocopies Duane-radial ray syndrome (also called Okihiro syndrome; OMIM #607323), which is caused by mutations in SALL4 (Asakawa and Kawakami, 2018; Cox et al., 2002; Kohlhase et al., 2005).

In murine cardiac and forelimb formation, Tbx5 regulates Sall4 expression, and coordinated positive and negative feed-forward loops between these two transcription factors are required for the correct patterning of the heart and limbs (Koshiba-Takeuchi et al., 2006). This relationship, at a molecular level, explains the shared phenotypes between patients with Duane-radial ray/Okihiro and thalidomide syndromes. Together with Holt-Oram syndrome phenotypes, these reflect the early developmental connection of the heart and forelimbs in the LPM.

Other examples of embryopathies linked to fetal teratogenic agent exposure include lithium-associated congenital heart defects and fetal retinoid syndrome. As retinoid acid signaling is involved in second heart field patterning, perturbations in this pathway cause heart malformations. The most common cause of fetal retinoid syndrome is exposure to isotretinoin, a retinoid derived from vitamin A that is used to treat cystic acne. An analysis of 154 patients with fetal exposure to isotretinoin concluded that there is a 35% risk of embryopathy when isotretinoin is taken beyond the 15th day post-conception (Lammer et al., 2010; Mondal et al., 2017). Isotretinoin exposure-associated congenital abnormalities include craniofacial and cardiac defects, as well as altered central nervous system development and thymic malformations (Lammer et al., 2010; Mondal et al., 2017). Mechanistically, isotretinoin increases apoptosis in cells of the cranial neural crest due to upregulation of FoxO3a (FOXO3) and TRAIL (TNFSF10) (Draghici et al., 2021; Ej, 1992; Gudas and Wagner, 2011; Niederreither et al., 2001). Further studies are required to tease out additional signaling players in isotretinoin-induced cardiac and neural crest defects during fetal development, and how the LPM is affected in these syndromes.

Lithium is a common treatment for bipolar disorders, acting to slow or halt the progression of neuronal loss through targets including neurotrophins, GSK3B and additional mitochondrial/endoplasmic reticulum-associated proteins (Machado-Vieira et al., 2009). Fetal lithium exposure is associated with Ebstein's anomaly, a right ventricular outflow tract defect: a comprehensive study found that infants exposed to lithium have an adjusted risk ratio of 1.65% for cardiac malformations relative to unexposed infants (Patorno et al., 2017). GSK3B inhibits canonical Wnt signaling (Dominguez et al., 1995; He et al., 1995; Klein and Melton, 1996; Peifer et al., 1994; Pierce and Kimelman, 1995; Stambolic et al., 1996; Yost et al., 1996), hinting at a possible mechanistic connection to the heart defects, as Wnt signaling contributes to several steps in cardiac patterning and morphogenesis (Cohen et al., 2007; Mandal et al., 2017; Martin et al., 2010; Schneider and Mercola, 2001; Ueno et al., 2007; Verhoeven et al., 2011). Nonetheless, lithium still remains the drug of choice to treat bipolar pregnant mothers due to evidence of efficacy compared to other drugs, as well as evidence that other drugs may also show low levels of teratogenicity (Patorno et al., 2017; Poels et al., 2018).

These examples underscore the risks of undesired effects from fetal exposure to environmental teratogenic agents. The modes of action of these drugs affect complex signaling pathways in different tissues and organs, but are often incompletely understood. Further, susceptibility to these environmental effects may greatly vary between patients due to unique modifying genetic variants, highlighting the need to integrate these factors when studying the mechanisms of syndromic diseases. The intertwined organ functions emerging from the LPM as well as its shared and co-deployed regulatory processes during development might render this mesoderm territory particularly sensitive to environmental perturbations.

Holt-Oram syndrome and Duane-radial ray syndrome, which we discuss in Box 3, present similar phenotypes due to genetic perturbations in a shared molecular pathway (Harvey and Logan, 2006): Tbx5 acts directly upstream of Sall4 during heart and appendage development in mice (Koshiba-Takeuchi et al., 2006) and in zebrafish (Harvey and Logan, 2006). Other heart-hand syndromes may result from developmental perturbation of the same or interacting pathways and the progenitor cell populations they control. Heart-hand syndrome type 3 (also known as Spanish type; OMIM #140450) features the characteristic upper limb deformities and congenital heart defects. Affecting three individuals of a single Spanish family, no causative gene has been assigned to the condition to date (Ruiz de la Fuente and Prieto, 1980). Additional orphan diseases that primarily present as congenital heart and limb defects include patent ductus arteriosus and bicuspid aortic valve with hand anomalies (OMIM #604381) (Gelb et al., 1999) and long-thumb brachydactyly syndrome (OMIM #112430) (Gelb et al., 1999; Hollister and Hollister, 1981) (Table 2). Despite the overlapping phenotypes, a shared progenitor cell or genetic origin has not been established in these orphan diseases. Therefore, investigating potential perturbations of TBX5, SALL4 and other cardiac/forelimb transcription factors in the LPM-derived heart and limb mesoderm warrants further consideration (Fan et al., 2009; Harvey and Logan, 2006; Koshiba-Takeuchi et al., 2006).

Numerous orphan diseases outside the heart-hand syndromes feature symptoms that affect LPM-derived organs or tissues. Examples include the previously mentioned WT limb-blood syndrome, with patients presenting endothelial, limb and craniofacial defects (Gonzalez et al., 1977), and thoracoabdominal syndrome (OMIM #313850), which affects craniofacial and neck structures, the heart, blood and kidneys (Carmi et al., 1990; Gonzalez et al., 1977). These clearly complex phenotypes could result from early perturbations in LPM patterning yet could also result from more pleiotropic developmental issues. Considering the joint LPM origin of various affected organs as the developmental stencil to narrow down possible connecting causes could advance the investigation of underlying mutations for these orphan diseases. Although orphan diseases are exceedingly rare, collectively, affected patients make up a large percentage of genetic diseases and deserve diagnostic and therapeutic investigation (Orphanet).

## Connecting and testing mechanisms of syndromic diseases using animal models

As outlined above, a LPM disease-associated gene may be expressed in multiple organ lineages. This is based on lineage connections or co-option of gene regulatory mechanisms during organ development in disparate cell types (McQueen and Rebeiz, 2020; Monteiro, 2012; Olson, 2006; Shubin et al., 2009). Throughout this Review, we have highlighted how model organisms have fundamentally contributed to our understanding of developmental birth defects, gene ontology and biological mechanisms (Fig. 3, Table 3). For example, the notion that head and neck muscles, together with the heart, are affected in DiGeorge syndrome indicates that they emerge from the Tbx1-expressing CPF (Diogo et al., 2015; Greulich et al., 2011; Lescroart et al., 2015; Swedlund and Lescroart, 2019). Lineage tracing experiments have established firm evidence for shared clonal origins of heart and head muscle lineages (Diogo et al., 2015; Lescroart et al., 2010, 2015; Mahadevan et al., 2004; Meilhac et al., 2004). Consequently, mutations in undifferentiated early CPF progenitors, such as mutations in *TBX1*, can potentially influence all downstream lineages. However, how Tbx1 is regulated in the CPF and whether its downstream expression in different muscle lineages uses shared or divergent mechanisms in each lineage remains to be clarified.

*TBX5*, as the causative gene in Holt-Oram syndrome, provides another potent example for joint expression in different LPM descendants. The recessive zebrafish mutant *heartstrings* carries a mutant *tbx5a* allele (Garrity et al., 2002). *heartstrings* mutants, as well as *tbx5a* morphants, display heart and pectoral fin defects, recapitulating the *Tbx5* mutant mouse and human Holt-Oram phenotypes (Ahn et al., 2002; Garrity et al., 2002) (Table 3). Studies in zebrafish *tbx5a* mutants and morphants have provided new insights into how Tbx5 contributes to left-right laterality, cardiac looping and myocardial conductivity (Boyle Anderson and Ho, 2018; Mao et al., 2015; Mao et al., 2021; Mosimann et al., 2015; Parrie et al., 2013). These insights provide basic concepts of the cardiac manifestations that could arise in patients carrying *TBX5* gene variants. Mice heterozygous for a *Tbx5* deletion (*Tbx5^del/+^*) recapitulate the hallmarks of the human syndrome with congenital forelimb and heart defects, whereas mice with homozygous deletions (*Tbx5^del/del^*) are embryonic lethal (Bruneau et al., 2001; Mori and Bruneau, 2004; Zhou et al., 2005). Mechanistic work revealed that Tbx5 and Nk2.5 synergistically interact to promote the expression of the gap junction protein Cx40 (Gja5), reduction of which is assumed to contribute to the cardiac anomalies in Holt-Oram syndrome (Bruneau et al., 2001; Zhou et al., 2005). In the limbs, absence of Tbx5 abrogates the Fgf10-Fgf8 signaling loop, which is critical for limb bud outgrowth (Agarwal et al., 2003; Arora et al., 2012; Minguillon et al., 2012). However, as we discussed earlier, whether Tbx5 activity in both cardiac and forelimb progenitors reflects a joint clonal LPM origin of these organ precursors, or whether Tbx5 expression and function are co-opted by forelimb precursors in a separate gene-regulatory event, remains unresolved. The mouse *Tbx5* locus harbors several regulatory elements, including a dedicated forelimb enhancer (Cunningham et al., 2018; Minguillon et al., 2012) and multiple cardiac enhancers (Richter et al., 2020; Smemo et al., 2012); more regulatory elements (and their upstream control) await discovery (Bickley and Logan, 2014; Bruneau et al., 1999). Similarly, zebrafish *hand2* and mouse *Hand2* mutants have provided key insights into the developmental mechanisms in heart, limbs and additional LPM organs influenced by this deeply conserved transcription factor (Barnes et al., 2011; Firulli et al., 1998; George et al., 2022; Hashimoto et al., 2019; Keegan et al., 2005; Laurent et al., 2017; McFadden et al., 2005; Osterwalder et al., 2014; Perens et al., 2016; Prummel et al., 2022; Vincentz et al., 2021; Yamagishi et al., 2000; Yelon and Stainier, 2005). The upstream regulation of Hand2 in mouse and zebrafish, beyond the pharyngeal arches (Charité et al., 2001; Iklé et al., 2012), remains vastly uncharacterized, as regulatory elements for broad early LPM expression or for heart and limb/pectoral fin activity have not been identified to date.

Multiple lineages may also develop in an interconnected manner, whereby perturbation of one may have consequences for another. Beyond Tbx5, the heart and the pectoral fin/forelimb also provide an example for inter-organ signaling interactions during development. In the absence of retinoic acid signaling in the prospective pectoral fin/forelimb field, the heart field expands in cell number (Cunningham and Duester, 2015; Duong et al., 2021; Gibert et al., 2006; Zhao et al., 2009). This interaction might reflect the evolutionary relationship between heart and forelimb, with models proposing that delimiting the heart field was a prerequisite to paired appendage evolution (Tanaka, 2013, 2016). Beyond the developmental impact of perturbing retinoic acid signaling and its upstream regulation, avoiding embryo or fetal exposure to retinoic acid analogs remains paramount (Box 3) (Cunningham and Duester, 2015; Duester, 2008; Lammer et al., 2010; Mondal et al., 2017; Niederreither et al., 2001). In the forming trunk, the kidney, hematopoietic and endothelial progenitors form in close proximity within the posterior LPM and are influenced by signaling interactions that include the lateralmost mesothelial progenitors (Fig. 1) (Chapman, 2019; Cheng et al., 2015; Dressler, 2009; Fleming et al., 2013; Perens et al., 2016; Shin et al., 2009; Wingert and Davidson, 2008). Consequently, perturbations of these developmental interactions could lead to multi-organ defects that manifest as complex syndromic phenotypes.

While work across animal models has discovered causes and consequences of congenital heart anomalies and their comorbidities, animal models also emphasize inter-species differences in genetic susceptibility to structural heart disorders. In humans, the majority of genetic congenital heart disease patients carry heterozygous mutations (Richards and Garg, 2010; Andersen et al., 2014; Nees and Chung, 2020; Pierpont et al., 2018; Williams et al., 2019; Yasuhara and Garg, 2021). By contrast, mouse and zebrafish models of these disorders frequently require bi-allelic loss of function of the orthologous gene(s) to reveal a phenotype, as documented for mutations in *Tbx5*, *Hand2*, *Sall4*, Gata family members and others (Bruneau et al., 2001; Garrity et al., 2002; Granados-Riveron et al., 2012; Harvey and Logan, 2006; Yelon et al., 2000) (Fig. 3, Table 3). Beyond complete loss of function, numerous mutations identified in patients result in amino acid substitutions or splicing and regulatory alterations with more subtle impact on gene function. Therefore, recapitulating the exact patient-derived mutation may be necessary to appropriately model a particular syndrome or patient cohort (Albadri et al., 2017; Kimura et al., 2014; Prykhozhij et al., 2018). Rather than reducing the validity of animal models, these nuances reveal the critical roles of gene dosage or of compensatory genetic mechanisms that await further elucidation (Rossi et al., 2015; Sztal and Stainier, 2020).

Taken together, the LPM provides a conceptual framework to connect seemingly disperse disease phenotypes, either by common clonal origin, close lineage connection during development or shared regulatory gene expression among progenitors. Considering developmental and molecular connections between cells and organs has vast potential in directing diagnostics for possible and plausible comorbidities, avoiding potential incomplete diagnoses, and facilitating the design of complex treatment strategies.

## Outlook

Next-generation sequencing has advanced ground-breaking insights into mutations and gene variants associated with developmental disorders and invigorated association studies for disease susceptibility loci (Austin-Tse et al., 2022; Lightbody et al., 2019; Morash et al., 2018; Yang and Kim, 2018). Several publicly available databases curate information on disease genetics, such as the Monarch Initiative and OMIM (McMurry et al., 2016; Shefchek et al., 2020; Motulsky, 1993) (Fig. 3). Although they are exceedingly powerful in aggregating diverse data from clinical and functional studies, any developmental or mechanistic connections for LPM-associated diseases, such as joint LPM origin of affected organs, remains to be determined by the database user. Further, drawing functional or causative conclusions from individual mutations or genetic variation is heavily influenced by insights gained in model organism studies (Alghamdi et al., 2022), in particular, assessing the phenotypic impact of individual gene perturbations on structural birth defects. As nowhere near all orthologs and paralogs of human genes have been functionally tested in developmental model organisms (Table 3, Box 2), the community still faces a gap in gene ontology knowledge that restricts the sequence-based prediction of disease traits. CRISPR-mediated mutagenesis for basic functional testing of candidate genes is possible by microinjection in zebrafish and *Xenopus*: these CRISPR-induced somatic mutants, so-called crispants (Burger et al., 2016), can recapitulate major aspects of the candidate gene's function. Nonetheless, although crispants provide a powerful discovery tool for first functional insights before mouse knockout studies, they require stringent controls and germline mutant validation in species that enable this workflow (Burger et al., 2016; Naert and Vleminckx, 2018; Shah et al., 2015; Trubiroha et al., 2018; Wu et al., 2018) (Fig. 3).

The broad lack of interfaces linking patient genetic data to functional *in vivo* follow-up, such as missing cross-species integration with animal models in individual databases, remains a key gap. The Model organism Aggregated Resources for Rare Variant ExpLoration (MARRVEL) website curates available model organism resources for human disease-relevant genes, providing a powerful resource for overcoming this gap (Wang et al., 2017a). Additional information, such as genomic coordinates, or interconnection with databases mapping non-coding features of the genome, could significantly expand patient-derived sequence data. Further, the varied versions of the human genome assembly used across individual databases renders comparisons challenging. Although individual genes of interest can be cross-referenced manually, the systematic generation of candidate gene lists to validate in model organisms remains labor intensive and not broadly accessible. Augmenting databases towards driving experimental validation of mutations, variants and causal gene discovery overall would provide fertile grounds for collaborations between clinicians, bioinformaticians and developmental biologists to accelerate research on rare LPM disorders, as we started to define throughout this Review, and beyond.

Ultimately, a major hurdle to diagnose and fully care for syndromic congenital anomalies remains the sheer complexity of human phenotypes such as heart-hand disorders. Patients with a specific organ-affecting disease often require highly specialized care. Performing a full diagnostic phenotype assessment of potentially dozens of symptoms in multiple organs is frequently not possible for reasons involving time, finances, technology or lacking consent. Nonetheless, providing insights into the underlying biology of linked comorbidities of syndromic congenital disease has the potential to improve long-term care and consequently the quality of life of affected patients. Predicting or anticipating care-relevant congenital phenotypes that can co-occur based on embryonic connections gleaned from developmental biology is only the starting point towards this goal.

## Supplementary Material

10.1242/dmm.049735_sup1 Supplementary information
