# Supplementary material for: Lateral thinking in syndromic congenital cardiovascular disease
Source: Dis Model Mech. 2023 May 3;16(5):dmm049735. doi: 10.1242/dmm.049735 (PMC10184679; doi:10.1242/dmm.049735)
Supplement: Supplementary information [file dmm-16-049735-s1.pdf]

Supplementary information

Steps to generate a list of OMIM searches, comparison table and Excel sheet with results.

1) Make an account on https://www.overleaf.com/ and upload the code below in a file.
2) Make a list in Excel with the diseases of interest - make a column with OMIM number (see example attached "list.csv").
3) Save this list as .csv (name it "list" for easiest use to preserve the current code).
4) Upload the table in overleaf and it will pop up in the compiled document.
5) Copy-paste the string on page 3 from the overleaf recompiled output in a browser.

<u>Example Overleaf interface:</u>

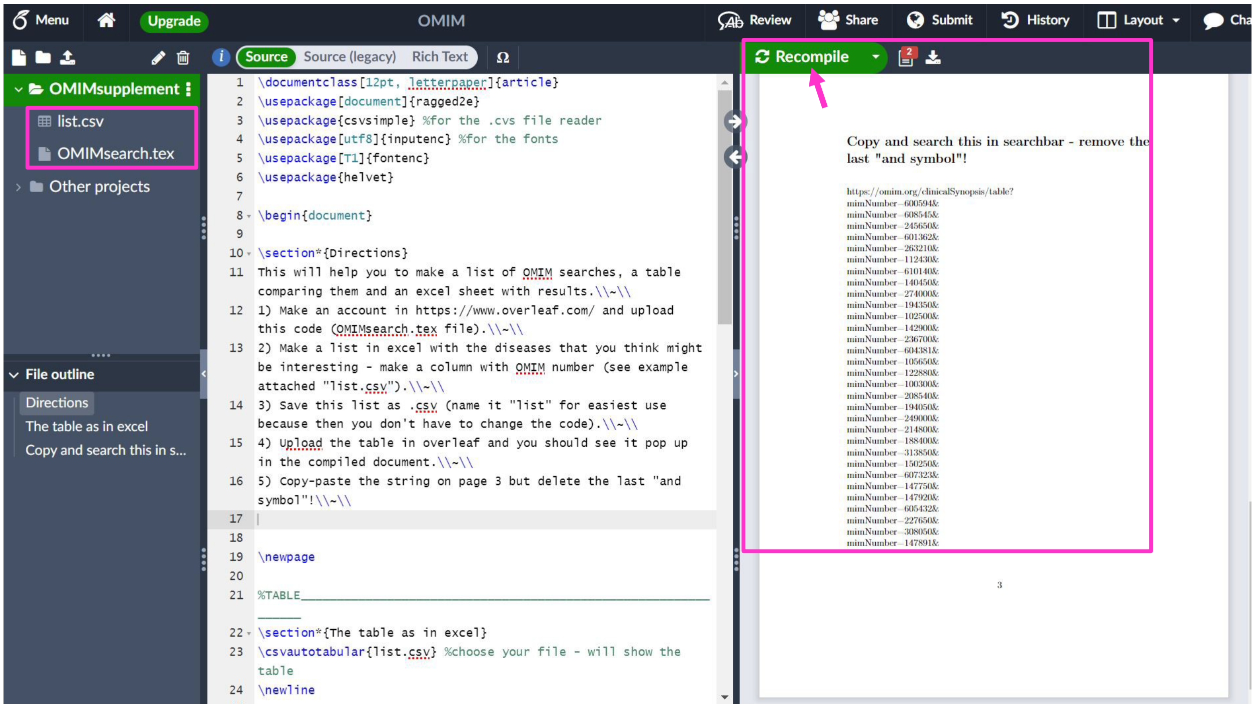

Code to upload in overleaf:

```
\documentclass[12pt, letterpaper]{article}
\usepackage[document]{ragged2e}
\usepackage{csvsimple} %for the .cvs file reader
\usepackage[utf8]{inputenc} %for the fonts
\usepackage[T1]{fontenc}
\usepackage{helvet}

\begin{document}

\section*{Directions}
This will help you to make a list of OMIM searches, a table comparing them
and an Excel sheet with results.\\~\\
1) Make an account on https://www.overleaf.com/ and upload this code
(OMIMsearch.tex file).\\~\\
2) Make a list in Excel with the diseases of interest - make a column with
OMIM number (see example attached
"list.csv").\\~\\
3) Save this list as .csv (name it "list" for easiest use to preserve the
current code).\\~\\
4) Upload the table in overleaf and it will pop up in the compiled
document.\\~\\
5) Copy-paste the string on page 3.


\newpage

%TABLE_______________________________________________________________
\section*{The table as in Excel}
\csvautotabular{list.csv} %choose your file - will show the table
\newline
~
\newpage

%COMPARISON TABLE____________________________________________________
\section*{Copy and paste this in a browser}\\~\\

\csvreader
[before first line = {https://omim.org/clinicalSynopsis/table?\\},
after line = \\]
{list.csv}{OMIM=\OMIM}
{mimNumber=\OMIM\&}


\end{document}
```

Disease Models & Mechanisms • Supplementary information

Example PDF output once run in overleaf:

Directions

This will help you to make a list of OMIM searches, a table comparing them and an Excel sheet with results.

1) Make an account on https://www.overleaf.com/ and upload this code (OMIMsearch.tex file).

2) Make a list in Excel with the diseases of interest - make a column with OMIM number (see example attached "list.csv").

3) Save this list as .csv (name it "list" for easiest use to preserve the current code).

4) Upload the table in overleaf and it will pop up in the compiled document.

5) Copy-paste the string on page 3.

Disease Models & Mechanisms • Supplementary information

The table as in excel

OMIM
600594
608545
245650
601362
263210
112430
610140
140450
274000
194350
102500
142900
236700
604381
105650
122880
100300
208540
194050
249000
214800
188400
313850
150250
607323
147750
147920
605432
227650
308050
147891

Copy and paste this in a browser

https://omim.org/clinicalSynopsis/table?
mimNumber–600594&
mimNumber–608545&
mimNumber–245650&
mimNumber–601362&
mimNumber–263210&
mimNumber–112430&
mimNumber–610140&
mimNumber–140450&
mimNumber–274000&
mimNumber–194350&
mimNumber–102500&
mimNumber–142900&
mimNumber–236700&
mimNumber–604381&
mimNumber–105650&
mimNumber–122880&
mimNumber–100300&
mimNumber–208540&
mimNumber–194050&
mimNumber–249000&
mimNumber–214800&
mimNumber–188400&
mimNumber–313850&
mimNumber–150250&
mimNumber–607323&
mimNumber–147750&
mimNumber–147920&
mimNumber–605432&
mimNumber–227650&
mimNumber–308050&
mimNumber–147891&
